# Cancer incidence following non-neoplastic medical conditions: a prospective population-based cohort study

**DOI:** 10.2340/1651-226X.2024.40757

**Published:** 2024-11-04

**Authors:** Lauri J. Sipilä, Tomas Tanskanen, Sanna Heikkinen, Karri Seppä, Mervi Aavikko, Janne Ravantti, Lauri A. Aaltonen, Janne Pitkäniemi

**Affiliations:** a Department of Medical and Clinical Genetics, University of Helsinki, Biomedicum Helsinki, Helsinki, Finland; b Applied Tumor Genomics, Research Programs Unit, University of Helsinki, Biomedicum Helsinki, Helsinki, Finland; c Finnish Cancer Registry, Helsinki, Finland; d Institute for Molecular Medicine Finland (FIMM), HiLIFE, University of Helsinki, Helsinki, Finland; e Molecular and Integrative Biosciences Research Programme, Faculty of Biological and Environmental Sciences, University of Helsinki, Finland; f Health Sciences Unit, Faculty of Social Sciences (Health Sciences), Tampere University, Tampere, Finland; g Department of Public Health, Faculty of Medicine, University of Helsinki, Helsinki, Finland

**Keywords:** Cancer epidemiology, comorbidity, lifestyle factors, cancer registry, public health

## Abstract

**Background and purpose:**

Many non-neoplastic diseases have been established to be tumorigenic, and cancers are sometimes misdiagnosed as non-neoplastic diseases. We conducted a comprehensive registry-based study of site-specific cancer diagnosis risk following the diagnosis of any preceding medical condition (PMC) encoded by the International Classification of Diseases (ICD)-10 classification.

**Material and methods:**

We analyzed healthcare data and cancer data for a random population-based sample of 2.5 million individuals living in Finland on January 1, 2000. Hazard ratios for each PMC and cancer pair were estimated using piecewise constant hazard regression models. *P*-values were corrected for multiple testing with the Bonferroni method.

**Results:**

Several lifestyle-related PMCs were associated with the risk of cancer diagnosis, exemplified by chronic obstructive pulmonary disease and subsequent lung cancer diagnosis risk (female hazard ratio [HR] = 9.91, 95% confidence interval [CI]: 9.18–19.7, *p*-adj. < 0.0001; male HR = 5.69, 95% CI: 5.43–5.96, *p*-adj. < 0.0001). Diagnosis risk of ill-defined cancers appeared to increase following diagnosis of Alzheimer’s disease (AD). We identified rare PMCs of potential interest.

**Interpretation:**

A considerable proportion of the statistically significant associations were explainable by tobacco smoking and alcohol use. The enrichment of ill-defined cancer diagnoses in persons with AD, together with the overall inverse association between AD and cancer, may reflect underdiagnosis of cancer in this patient population. Our results provide a useful resource for research on the prevention and early detection of cancer.

## Introduction

Cancer incidence is known to be affected by several preceding diseases. Genetic disorders such as Lynch syndrome, Li-Fraumeni syndrome, and neurofibromatosis type 1 (NF1) severely increase the lifetime cancer risk of the patients. Viral and bacterial infections are also major contributors to cancer incidence worldwide [1]. Chronic viral hepatitis and diabetes mellitus are associated with an increased risk of liver cancer [2, 3], exemplifying how disparate diseases may promote the same cancer. The symptoms of cancer may initially be attributed to a benign condition. For example, rectal bleeding due to colorectal cancer may be attributed to hemorrhoids. On the other hand, cancer may be the underlying cause of another disease such as diabetes mellitus or a paraneoplastic syndrome [4]. Environmental agents can cause both cancers and noncancerous diseases, with tobacco smoking being a significant cause of not only cancer but also of various cardiovascular, metabolic, respiratory, and digestive diseases [5]. Preceding medical conditions (PMCs) may also affect the likelihood of being diagnosed with existing cancer. For instance, individuals with schizophrenia are more likely to die of previously undiagnosed cancer compared to the general population [6].

Large-scale health data is an increasingly important source of information in biomedical research. It allows the assessment of multiple research questions without the time- and cost-intensive process of prospective data collection. For example, in the Prospective Meta-Cohort Study of Cancer Burden in Finland (METCA project), the Finnish Institute for Health and Welfare and Finnish Cancer Registry (FCR) have combined large national health studies to evaluate population attributable fractions for cancer risk factors [7, 8]. In Sweden, the associations of blood group antigens and 1,217 disease endpoints were studied in a sample of 5.1 million individuals by linking a blood donation and transfusion database to national health registries [9]. A sample from The Danish National Patient Register, covering 7.2 million individuals and 1,777 International Classification of Diseases (ICD)-10 disease codes, has been used to generate a browser of disease trajectories [10]. Here, we have linked FCR data with the Finnish Care Register for Health Care and conducted a comprehensive study of associations between non-neoplastic PMCs and the subsequent risk of cancer diagnosis. This is, to our knowledge, the first study using large-scale hospital data and complementary cancer registry data for a thorough scan of population-based cancer risk following almost any condition or event encoded by the ICD, 10th Revision (ICD-10).

## Material and methods

The study cohort consisted of a random population-based sample of 2.5 million individuals (Table 1). Study participants were sampled from the Population Information System maintained by the Digital and Population Data Services Agency (https://dvv.fi/en/individuals). All individuals with a permanent address in Finland and alive on 01 January 2000 were eligible for the sampling regardless of age, with the sample representing roughly half of the population of Finland at January 1, 2000. This was also the date of cohort entry for all participants. For each analyzed combination of PMC and cancer site, follow-up started on January 1, 2000 and ended at site-specific cancer diagnosis, death, emigration or December 31, 2017, whichever occurred first (Table 1).

**Table 1:** Numbers of observations and follow-up time in person-years for different end-of-follow-up states and any primary cancer diagnosis by gender.

Study subjects and events	Female		Male	
	Number	Person-years	Number	Person-years
Individuals	1,264,023	20,114,346	1,218,067	19,381,486
Death during follow-up	221,993	2,111,862	225,334	2,122,001
Emigration during follow-up	39,636	351,679	33,235	277,820
End of follow-up	949,132	17,081,148	915,522	16,476,282
New primary cancer during follow-up	109,125	1,028,483	113,089	1,069,346

Data presented for individuals with no previous cancer diagnoses at the start of follow-up.

PMC data were acquired from the Care Register for Health Care and included individual-level data on hospital discharges in 1996–2017 and outpatient clinic visits in 1998–2017. Complete records of all diagnoses and procedures for all study participants totaled 137 million entries. Diagnosis codes were based on the international ICD-10 classification with minor national modifications. The most significant difference between the Finnish and the international version of ICD-10 is lesser detail in the classification of external causes of morbidity and mortality (ICD-10 chapter XX) [11]. Cancer diagnoses of all participants in 1953–2017 were obtained from the FCR, and first primary cancers diagnosed after January 1, 2000, were used in the analysis. Cancers were classified into 61 cancer types based on the official cancer classification of the FCR, which is largely equivalent to ICD-10 (Table 2) [12, 13].

**Table 2:** Numbers of incident primary cancers occurring during follow-up in the study cohort.

Cancer	Abbreviation	ICD-10	Number of incident cancers, female	Number of incident cancers, male
Any		C00-96, D09.0-1, D32-33, D41-43, D45-47, D76	116,335	118,103
Any hematological*		C81-96, D45-47, D76	10,589	11,692
Acute lymphoblastic leukemia/lymphoma	ALL	C91.0	178	245
Acute myeloid leukemia	AML	C92.0	769	809
Adrenal gland	Adrenal	C74	87	87
Anus		C21	218	148
Bladder and urinary tract	Urinary	C65-68, D09.0-1, D41.1-9	2,188	7,251
Bone		C40–41	163	166
Brain		C71,D33.0-2,D43.0-2	1,644	1,920
Breast		C50	38,013	180
Cervix uteri	Cervical	C53	1,351	<5
Colon and rectum	CRC	C18–20	11,527	12,169
Corpus uteri	Uterine	C54	7,027	<5
Cranial nerves	Cranial n.	C72.2–5, D33.3, D43.3	255	220
Digestive organs, other, and unspecified	Digestive	C26	543	381
Eye		C69	208	226
Female genital, other, and unspecified	Genital	C55, C57.5–9	463	<5
Gallbladder, bile ducts	Biliary	C23–24	1,457	879
Hodgkin lymphoma	HL	C81	550	674
Ill-defined or unknown	Ill-defined		2,876	2,181
Kidney		C64	3,223	4,038
Larynx, epiglottis	Laryngeal	C32	111	906
Leukemia, other, or unspecified		C95	230	225
Lip		C00	270	478
Liver		C22	1,402	2,360
Lung, trachea	Lung	C33–34	6,803	14,812
Male genital, other, and unspecified	Genital	C63	<5	36
Mature B cell neoplasms	Mat. B		5,528	6,500
Mature T and NK cell lymphomas/leukemias	Mat. T&NK	C84	327	442
Melanoma of the skin	Melanoma	C43	4,792	5,066
Meninges		C70,D32,D42	2,733	921
Mesothelioma	Mesoth.	C45	168	608
Mouth, other, or unspecified	Mouth	C03–06	564	655
Myelodysplastic syndromes and myelodysplastic/myeloproliferative neoplasms	MDS		526	567
Myeloproliferative neoplasms	Myelopr.	C92.1, D45, D47.1, D47.3	1,184	1,067
Non-Hodgkin lymphoma, other, or unspecified	Non-HL	C85	1,421	1,353
Nose, sinuses	Sinonasal	C30–31	158	212
Oesophagus		C15	752	1,586
Other endocrine glands	Endocrine	C75	28	37
Other or unspecified respiratory or intrathoracic organs	Respiratory	C37–39	151	159
Other, unspecified, or mixed hematological disease	Blood	C96, D76	48	35
Ovary, etc.		C56, C57.0–4, C48.1–2 Serous)	4,279	<5
Pancreas		C25	4,833	4,505
Penis		C60	<5	253
Peripheral nerves, autonomic nervous system	PNS	C47	29	41
Pharynx		C01, C09–14	328	1,034
Placenta		C58	23	<5
Prostate		C61	<5	40,707
Salivary glands	SG	C07–08	278	260
Skin, other	Skin	C44 (Other)	526	530
Skin, squamous cell carcinoma	Skin SCC	C44 (Squamous cell)	5,087	5,330
Small intestine	SI	C17	442	535
Soft tissues		C48–49	931	762
Spinal cord		C72.0–1,D33.4,D43.4	159	153
Stomach		C16	2,643	3,327
Testis		C62	<5	1,090
Thyroid gland	Thyroid	C73	2,760	907
Tongue		C02	471	551
Unspecified central nervous system	CNS	C72.8–9, D33.7–9, D43.7–9	21	15
Vagina		C52	159	<5
Vulva		C51	741	<5

Some individuals were diagnosed with multiple primary cancers during follow-up. Some presented abbreviations are only used to improve the readability of graphs where applicable. Exact numbers of observations are presented only for cancer-and-gender combinations with at least five observations. *=aggregated class consists of acute lymphoblastic leukemia/lymphoma, acute myeloid leukemia, Burkitt’s lymphoma/leukemia, chronic lymphatic leukemia, chronic myeloid leukemia, diffuse B lymphoma, essential thrombocythemia, follicular B lymphoma, histiocytic and dendritic cell neoplasms, Hodgkin lymphoma, mantle cell lymphoma, marginal zone lymphoma, mastocytosis, myelodysplastic syndromes, myelofibrosis, myeloma and other plasma cell tumors, mature T-cell neoplasms of the skin, myelodysplastic/myeloproliferative neoplasms, polycythemia vera, and other or mixed hematological neoplasia.

PMCs were defined as the exposure variable in our statistical model. We analyzed the data using the second and third hierarchy levels of the national ICD-10 classification, which are generally equivalent to the three-character category codes (ICD-10–3c) and four-character subcategory codes (ICD-10–4c) of the international ICD-10. With both levels combined, a total of 9,998 disease entities were analyzed. Thus, the PMC data consisted of 24,986,722 unique pairs of individuals and diagnoses on the ICD-10-3c level (10,607,379 in men and 14,379,343 in women) and 26,352,387 on the ICD-10-4c level (11,118,517 in men and 15,233,870 in women). Combinations of ICD-10 codes containing information about both an underlying generalized disease and a manifestation in a particular organ or site were considered as separate codes. For example, the code for influenza-related encephalitis G05.1*J09 was split into the codes G05.1 and J09. The outcome variable was diagnosis of a primary cancer during the follow-up period (Table 2).

Exposure and outcome data were linked by the unique personal identity code assigned to all Finnish citizens and permanent residents. There was a total of 96,502 PMC-cancer type combinations on the ICD-10-3c level and 513,376 on the ICD-10-4c level. Individuals with multiple primary cancers or PMCs contributed to all applicable PMC-cancer pair analyses. PMC status was determined by the time elapsed since PMC diagnosis. PMC diagnosis, and thus the beginning of exposure, was recorded even if it occurred before the start of follow-up on January 1, 2000. Individuals with prevalent cancer at the start of follow-up were excluded from the analysis of the same cancer site but permitted to contribute to analyses of different cancer types.

To study the risk of cancer diagnosis following PMCs, hazard ratios (HRs) with 95% confidence intervals (CIs) were estimated using piecewise constant hazard regression models with Poisson likelihood. The estimates were adjusted for attained age (0**–**4, 5**–**9, …, 90**–**94, or ≥ 95 years) and calendar period (2000–2005, 2006–2011, or 2012–2017). PMC status was allowed to vary over time and was categorized into three strata: no diagnosis of PMC, less than 1 year after PMC diagnosis, or at least 1 year after PMC diagnosis. The analyses were performed separately by gender.

To mitigate reverse causality and identify conditions with long-term tumorigenic significance, we report model-based HRs for individuals exposed to PMCs for at least 1 year. We filtered out results for ICD-10 codes of neoplasms (C00–C97, D00–D48) and symptoms, signs, and abnormal clinical and laboratory findings (R00–R99) due to their expected associations with subsequent cancer diagnosis. Lastly, to protect individual participants from being identified from the results, and to limit the reporting of spurious associations, the main results table includes only PMC-cancer diagnosis risk associations in which at least five individuals have developed the relevant cancer. After filtering, the final number of PMC-cancer risk associations was 73,295. P-values were adjusted for all ICD-10-3c and ICD-10-4c level analyses passing filtering criteria (N = 73,295) across both men and women combined, using both Bonferroni correction and the Benjamini-Hochberg procedure with a false discovery rate (FDR) of 0.1. For some PMCs, the Finnish ICD-10 classification did not provide an English translation, and we have left these untranslated.

To visualize the spectrum of PMC-cancer risk associations, we generated a Manhattan plot of all category-level ICD-10 codes and cancers. A hierarchical cluster analysis of binary logarithms of HR point estimates was conducted using Euclidean distance as the distance metric and Ward linkage as the linkage criterion. Clusters were estimated for a subset of the data consisting of cancer sites with at least 100 ICD-10-3c code associations passing the filtering criteria and ICD-10-3c codes with an HR estimate for at least 30 or 33 cancer sites in men or women, respectively. The thresholds for cancer site numbers were chosen iteratively to maximize the number of data points in the cluster analysis while minimizing missing values. For less common diseases, the spectrum of cancers that passed the filtering criteria was too limited to be meaningfully incorporated into the hierarchical cluster analysis.

We used the R packages data.table version 1.12.2 and popEpi version 0.4.10 to analyze the data, ggplot2 version 3.4.0 to generate the Manhattan plot, and gplots version 3.1.3 to generate the hierarchical clustering plots.

## Results

**Figure 1:**
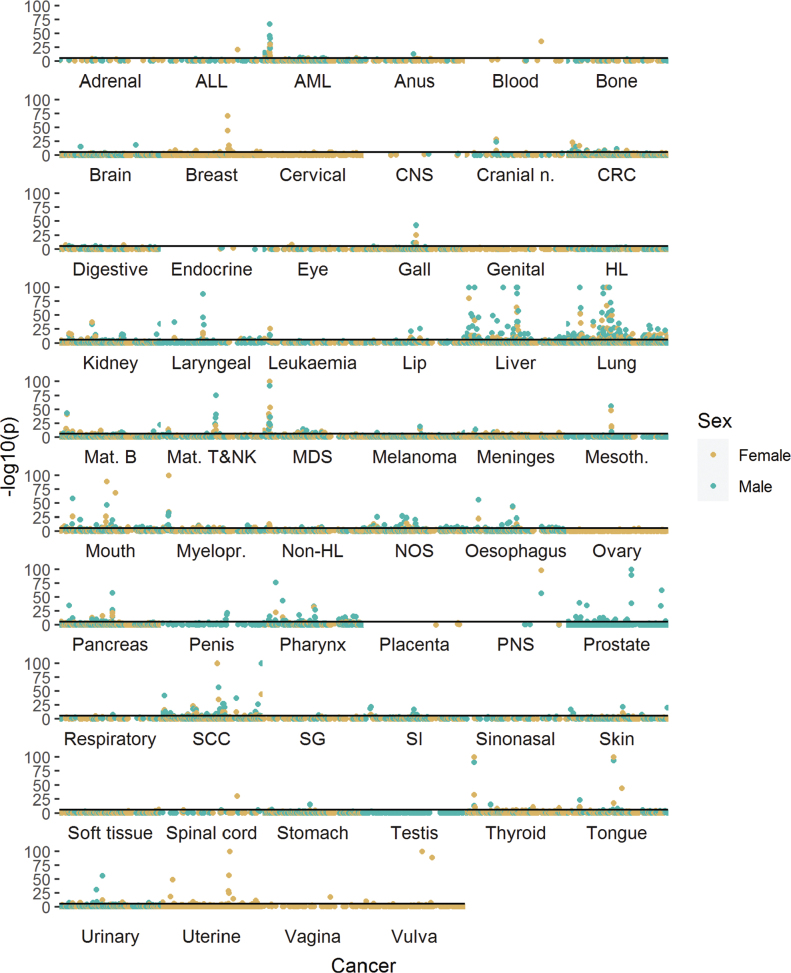
Manhattan plot of preceding medical conditions (PMCs) at the three-character level of ICD-10 and cancer diagnosis risk for each cancer site for women (A) and men (B). Complete results are available in the Supplementary material. Sorted ICD-10-codes (x-axis) and log-transformed *p*-values (y-axis) are presented, with each point signifying a PMC-cancer pair. Point color alternates between sites. Solid horizontal lines denote a Bonferroni-adjusted *p*-value of 0.05. Y-axis values are cut at negative log10 value of 100. An abbreviation key is presented in Table 2.

**Figure 2:**
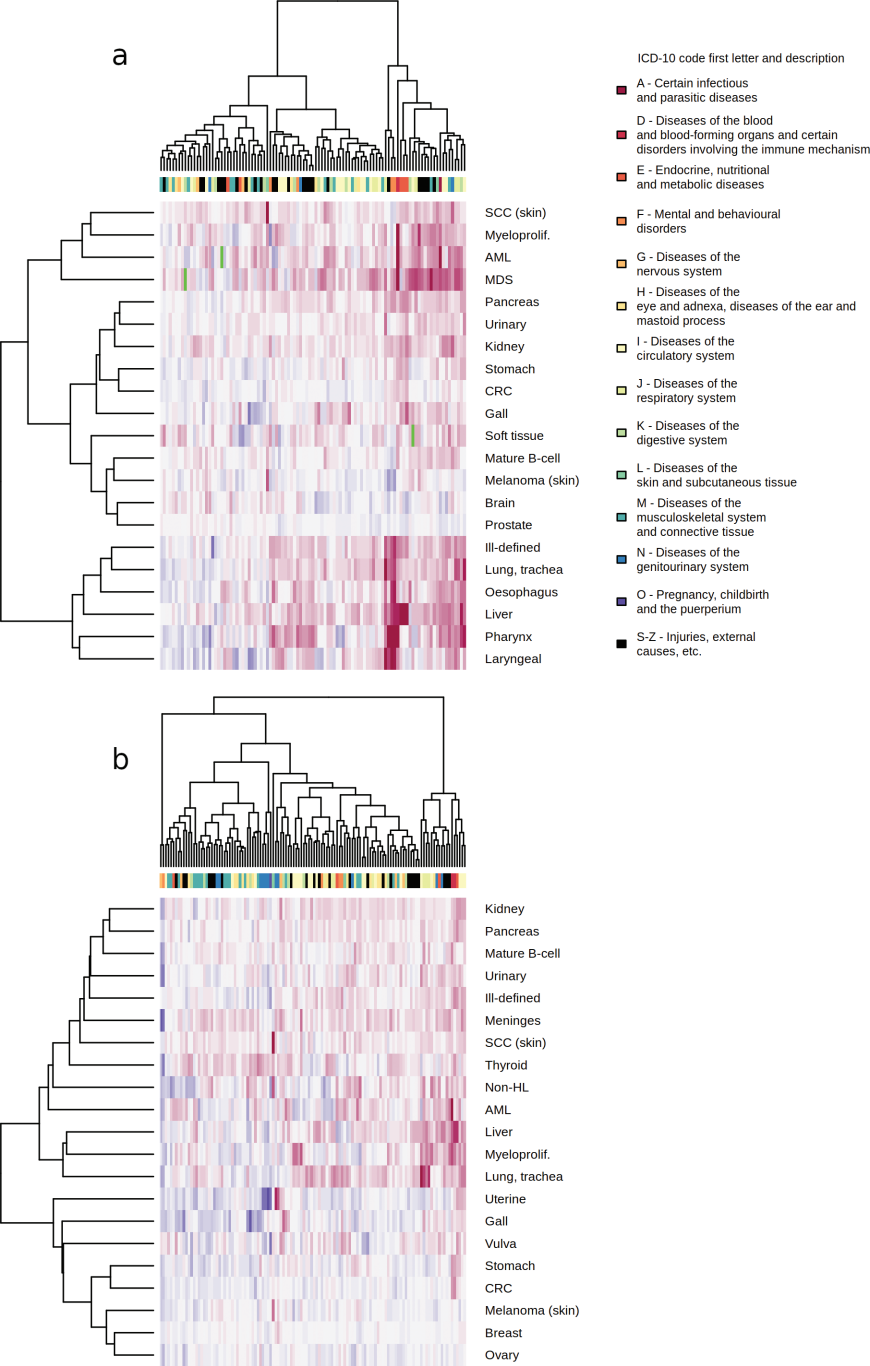
Hierarchical clustering of binary logarithms of common three-character level ICD-10 code (columns) and cancer (rows) hazard ratio estimates, with blue color indicating a decreased cancer diagnosis risk, red indicating an increased cancer diagnosis risk, and green indicating a missing data point. Men in image A, women in image B.

A table of 73,295 associations of PMCs and cancers occurring at least 1 year from PMC diagnosis was produced (Supplementary material). Cancer sites with a strong etiological link to either tobacco smoking or alcohol use showed a larger number of statistically significant associations with PMCs than most other sites in both men and women (Figure 1). The most significant PMC associated with lung cancer risk was chronic obstructive pulmonary disease (ICD-10 J44, female HR = 9.91, 95% CI = 9.18–19.7, Bonferroni-adjusted *p* < 0.0001; male HR = 5.69, 95% CI: 5.43–5.96, *p*-adj < 0.0001). For many PMC-cancer combinations, the association appeared to be predominantly explained by tobacco smoking. There was also a high risk of lung cancer following diagnosis of ‘mental and behavioral disorders due to use of tobacco’ (F17, female HR = 9.29, 95% CI: 6.99–12.35, *p*-adj. < 0.0001; male HR = 5.37, 95% CI: 4.34–6.64, *p*-adj. < 0.0001). In liver cancer, diseases of the liver (K70–77) formed a peak of consecutive high-significance associations in the Manhattan plots, while esophageal varices were most significantly associated with liver cancer in both men and women (ICD-10 I85, male HR = 56.68, 95% CI: 47.19–68.09, *p*-adj. < 0.0001; female HR = 85.61, 95% CI: 64.85–113.01, *p*-adj. < 0.0001). PMCs typically associated with either tobacco or alcohol use had overlapping patterns of cancer risk, with lung cancer clustering together with alcohol-related cancers in men (Figure 2A), while a similar but less pronounced trend was present in women (Figure 2B). Similar associations were also observed following certain injury diagnoses, where fracture of neck of femur (ICD-10 S72.0) increased the diagnosis risk of lung cancer in both genders (male HR = 2.01, 95% CI: 1.76–2.29, *p*-adj. < 0.0001; female HR = 1.76, 95% CI: 1.52–2.05, *p*-adj. < 0.0001) and diagnosis risk of pharyngeal cancer in men (HR = 3.88, 95% CI: 2.39–6.29, *p*-adj. = 0.001**;** female HR = 3.95, 95% CI: 1.97–7.91, *p*-adj. = 1). Physical activity-related injuries showed inverse associations; for example, internal derangement of knee (ICD-10 M23) was associated with decreased lung cancer diagnosis risk in men (HR = 0.68, 95% CI: 0.61–0.75, *p*-adj. < 0.0001**;** female HR = 0.89, 95% CI: 0.78–1.01, *p*-adj. = 1), and ‘pedal cyclist injured in transport accident’ (ICD-10 V10–V19) was nonsignificantly associated with decreased risk of any cancer in women (HR = 0.87, 95% CI: 0.8–0.95, *p*-adj. = 1**;** male HR = 1.09, 95% CI: 1.02–1.17, *p*-adj. = 1).

PMCs affecting cognitive function decreased cancer diagnosis risk in a wide range of sites. Alzheimer’s disease (AD, ICD-10 G30) and dementia (ICD-10 F00-03) were inversely associated with most of the analyzed cancer types (Supplementary table). Following AD diagnosis, diagnosis risk decreased statistically significantly for colorectal cancer (female HR = 0.67, 95% CI: 0.59–0.77, *p*-adj. < 0.0001; male HR = 0.6 95% CI: 0.50–0.71, *p*-adj. = 0.0003), mature B-cell neoplasms (female HR = 0.51 95% CI: 0.41–0.63, *p*-adj. < 0.0001; male HR = 0.48, 95% CI: 0.36–0.62, *p*-adj. = 0.004), female lung cancer (HR = 0.51, 95% CI: 0.41–0.63, *p*-adj. < 0.0001; male HR = 0.77, 95% CI: 0.66–0.89, *p*-adj. = 1), prostate cancer (HR = 0.50, 95% CI: 0.44–0.55, *p*-adj. < 0.0001), and female urinary cancer (HR = 0.39, 95% CI: 0.28–0.55, *p*-adj. = 0.003; male HR = 0.75, 95% CI: 0.62–0.90, *p*-adj. = 1). Dementia had a largely similar spectrum of statistically significant risk decreases. We observed a reversal of this trend to increased risks of unspecified or ill-defined cancer diagnosis although none of these associations remained statistically significant after correcting for multiple testing. Unspecified digestive organ cancer in women (G30, HR = 1.56, 95% CI: 1.15–2.13, *p*-adj. = 1; male HR = 1.33, 95% CI: 0.77–2.30, *p*-adj. = 1) was the most notable example. Schizophrenia (ICD-10 F20) was associated with an increased diagnosis risk of lung cancer (male HR = 2.96, 95%CI: 2.61–3.36, *p*-adj. < 0.0001; female HR = 2.89, 95% CI: 2.45–3.41, *p*-adj. < 0.0001) and female breast cancer (HR = 1.37, 95% CI: 1.24–1.51, *p*-adj. < 0.0001; male HR = 1.67, 95% CI: 0.41–6.76, less than five observations), while prostate cancer diagnosis risk was reduced (HR = 0.50, 95% CI: 0.42**–**0.59, *p*-adj. < 0.0001).

The results for rare PMC-cancer pairs and PMCs with weak-to-moderate associations with cancer diagnosis risk may reveal novel insights. For example, diagnosis of Lyme disease (ICD-10 A69.2) was associated with an increased risk of brain cancer diagnosis in women (HR = 3.55, 95% CI: 1.69–7.46, *p*-adj. = 1; male HR = 1.01, 95% CI: 0.25–4.04, less than five observations) and urinary cancer diagnosis in men (HR = 1.94, 95% CI: 1.24–3.04, p = 0.002, *p*-adj. = 1; female HR = 0.68, 95% CI: 0.17–2.74, less than five observations). For psoriasis (ICD-10 L40), the highest risk increase was noted for male breast cancer (HR = 3.47, 95% CI: 1.53–7.84, *p* = 0.001, p-adj. = 1; female HR = 0.99, 95% CI: 0.88–1.1, *p*-adj. = 1). Diagnosis of ‘other congenital malformations of face and neck’ (ICD-10 Q18) increased diagnosis risk of cervical cancer (HR = 4.33, 95% CI: 1.8–10.42, *p*-adj. = 1), lung cancer (female HR = 2.46, 95% CI: 1.28–4.73, *p*-adj. = 1; male HR = 1.61, 95% CI: 0.93–2.77, *p*-adj. = 1) and female kidney cancer (HR = 3.03, 95% CI: 1.26–7.29, *p* = 0.006, *p*-adj. = 1; male HR = 1.15, 95% CI: 0.37–3.58, less than five observations), while its subcategory ‘sinus, fistula, and cyst of branchial cleft’ (ICD-10 Q18.0) increased diagnosis risks for any cancer (male HR = 1.52, 95% CI: 1.14–2.02, *p*-adj. = 1; female HR = 1.16, 95% CI: 0.84–1.6, *p*-adj. = 1) and lung cancer (male HR = 2.6, 95% CI: 1.44–4.70, *p*-adj. = 1; female HR = 2.24, 95% CI: 0.84–5.97, less than five observations). Some combinations produced surprising inverse associations; for example, emphysema (ICD-10 J43) was associated with a decreased risk of female breast cancer diagnosis (HR = 0.44, 95% CI: 0.23–0.85, *p* = 0.007, *p*-adj. = 1).

## Discussion

We conducted a comprehensive study of population-wide healthcare data and estimated 73,295 gender-specific HRs for cancer diagnosis in persons with pre-existing medical conditions. We used high-quality nationwide health register data [14] from roughly half of the population of Finland. The large sample size allows the quantification of cancer diagnosis risk in common comorbidities and enables the detection of rarer and possibly previously unknown PMC-cancer associations. Knowledge of links between non-neoplastic medical conditions and cancers may propel further research on the prevention and early detection of cancer, potentially leading to better outcomes. Here, we report examples of both previously well-known PMC-cancer pairs and potentially novel associations. Associations presented in the main table of results (Supplementary material) should be viewed critically since despite analyzing high-quality registry data, we lack information on possible confounders beyond age and gender.

We present Bonferroni-corrected *p-*values to address the multiple comparisons problem and identify highly significant associations. Due to the conservativeness of the Bonferroni method, the Benjamini-Hochberg procedure may be preferred when studying associations between rare PMCs and cancers. We provide both values in our complete results (Supplementary material).

Various local conditions affect disease incidence. These include the environment, population genetics, society, and culture [15, 16]. Consequently, the statistical power of the study is likely to depend on these local conditions, and the degree of confounding may also depend on the study population. The Finnish Care Register for Health Care includes primary healthcare data only since 2011, and therefore we restricted the PMC records to specialized healthcare [14]. The use of single ICD-10 codes to define PMCs may have led to some degree of exposure misclassification because patients with related diagnostic codes, especially at the ICD-10-4c level, may share similar pathophysiological characteristics. Misclassification may also occur if an individual has diagnoses for a PMC only before the year 1996.

In the cohort study design, HRs measured the risk of subsequent cancer diagnosis following a PMC code, and the observed associations may be explained by various types of phenomena. While the study design is agnostic to the type of association observed, we hypothesize that there may be five common causes of association: (1) an undiagnosed cancer or a precancerous condition causing symptoms; (2) a PMC directly promoting tumorigenesis; (3) an environmental or behavioral factor confounding the analysis by causing both the PMC and cancer; (4) a PMC affecting the likelihood of an existing cancer being detected; and (5) possible competing risks of death.

Some PMCs may represent symptoms of underlying cancer, and the true sequence of events may in fact be the inverse of the observed one [4]. In our results, this is likely exemplified by the high risk of being diagnosed with mature T-cell lymphoma after a diagnosis of parapsoriasis or atopic dermatitis. Both may represent a misdiagnosis of mature T-cell lymphoma [17, 18]. Similarly, dorsopathy codes were associated with a spectrum of cancer diagnoses, and back pain is sometimes the first symptom of cancer [19].

In many instances, a high HR reflects a true risk of a tumorigenic process driven by the PMC, as exemplified by Crohn’s disease and small intestinal cancer, in which the cancer may have different characteristics depending on whether it manifests in the context of Crohn’s disease or *de novo* [20].

We present selected examples of potentially novel PMC-cancer associations. The association between Lyme disease and brain cancer in women is interesting because of the known neurological manifestations of Lyme disease. We observed an increased risk of male breast cancer diagnosis following a psoriasis diagnosis. An increased prevalence of male breast cancer in patients with psoriasis has been previously reported in a Swedish study and was suspected to result from a multiple comparisons problem [21], whereas a Danish study did not observe any statistically significant difference [22]. Finally, congenital malformations of the face and neck (ICD-10 Q18) were associated with a variety of cancers. This category might include persons with rare congenital branchio-oto-renal or branchio-otic syndromes, for which we are not aware of a previously known role in cancer predisposition. This finding might also be explained by increased frequency of head and neck imaging in individuals with related cancer, leading to an increased rate of branchial cleft cyst diagnosis compared to the general population.

Environmental agents may predispose to both cancer and noncancerous disease. In the hierarchical cluster analysis, we observed a subset of cancers clustering together in men based on their shared spectrum of risk-increasing PMCs. We observed PMCs predominantly related to alcohol use increasing diagnosis risk of lung cancer and PMCs related to tobacco smoking increasing liver cancer diagnosis risk. This indicates potential confounding in which excess cancer cases have been contributed by individuals who have had both exposures concurrently. Such individuals may contribute, for example, to the risk increases of lung cancer diagnosis following the diagnosis of alcoholic liver disease (ICD-10 K70, female HR = 4.78, 95% CI: 3.61–6.34, p-adj. < 0.0001; male HR = 2.29, 95% CI: 1.9–2.76, p-adj. < 0.0001), as investigations focused on the connection of alcohol use and lung cancer have observed only modest [23] or no [24] risk increase.

PMCs potentially affecting the likelihood of cancer diagnosis is exemplified by AD. A decreased cancer risk following AD and dementia has been observed in numerous studies [25], with both statistical and biological phenomena discussed as potential causative factors. The decreased cancer risks in our study are consistent with previously published results on colorectal cancer [26, 27], prostate cancer [26, 27], and lung cancer [26]. Of note, we observed increased HRs for ill-defined cancer diagnoses following diagnosis of AD, which may reflect challenges in diagnosing cancer in patients with AD. However, such increased risks were not statistically significant after adjusting for multiple testing. These observations may be attributable to individuals with AD not being able to consent to and partake in extensive diagnostic processes, leading to both fewer cancer diagnoses overall and an excess of ill-defined diagnoses. In schizophrenia, an increased mortality risk related to breast, colon, and lung cancer has been described elsewhere [28–30], which is in line with our results of increased diagnosis risks for lung and breast cancer. The dramatic increase in lung cancer risk is likely a result of heavy smoking among patients with schizophrenia [31], while the associations with breast and prostate cancer have been suggested to be related to adverse effects of antipsychotic medication [29, 30].

We observed some surprising inverse associations in some PMCs, especially with prostate and breast cancer. We presume that at least some of these risk decreases are caused by comparatively more cancer diagnoses being made shortly after PMC diagnosis. In the example of decreased breast cancer diagnosis risk following the diagnosis of emphysema, cancer may be diagnosed during the diagnostic process of the PMC. Thus, there may be an increased risk of breast cancer diagnosis in the first year after emphysema diagnosis and an inverse association thereafter. We cannot rule out the possibility that the competing risk of death may explain the inverse association between emphysema and breast cancer.

In conclusion, we have estimated cancer diagnosis risks following non-neoplastic PMCs by linking FCR data with the Finnish Care Register for Health Care and interpreted both general trends in the data and specific pairs of PMCs and cancers. The presented results are based on modeling in an hypothesis-agnostic framework. Consequently, the associations may lack adjustment for relevant unavailable factors, such as smoking, and may be affected by unmeasured confounding. Despite the large sample size, meaningful associations between rare exposures and rare cancers may have been missed due to the number of observations still being too low. We have shown that the results both agree with and refine existing knowledge and reveal potentially novel associations to be validated in other studies. Fewer or ill-defined cancer diagnoses in patients with AD and dementia underscore a need to evaluate healthcare in these patient populations. Our results provide a useful resource for research on the prevention and early detection of cancer.

## Declarations

### Author contributions

TT, SH, KS, LAA, and JP conceived and designed the study. LJS and JR procured computing resources. LJS, TT, and KS carried out formal analysis. All authors analyzed the results. LJS prepared visualizations. LJS wrote the initial draft; all other authors reviewed and edited. SH, LAA, and JP supervised the project. All authors reviewed and approved the final manuscript.

### Conflict of interest statement

The authors declare no conflict of interest.

### Ethics declarations

An ethics committee approval is not required for registry-based studies in Finland according to the Act on Secondary Use of Health and Social Data (552/2019). This study has been approved by the Finnish Institute for Health and Welfare (THL/118/6.02.00/2019) and the Digital and Population Data Services Agency (VRK/4504/2019-2).

### Data availability statement

The analyzed raw data cannot be made available by the researchers, due to research permits restricting the sharing of data on ethical and legal grounds. Permissions to use similar administrative health data from various register keepers can be applied from Findata, the Social and Health Data Permit Authority: https://findata.fi/en/.

## Supplementary Material

Cancer incidence following non-neoplastic medical conditions: a prospective population-based cohort study
